# Potential of Electrospun Nanofibers for Biomedical and Dental Applications

**DOI:** 10.3390/ma9020073

**Published:** 2016-01-26

**Authors:** Muhammad Zafar, Shariq Najeeb, Zohaib Khurshid, Masoud Vazirzadeh, Sana Zohaib, Bilal Najeeb, Farshid Sefat

**Affiliations:** 1 Department of Restorative Dentistry, College of Dentistry, Taibah University, Madina Munawwarah 41311, Saudi Arabia; 2 Department of Restorative Dental Sciences, Al Farabi Colleges, King Abdullah Road, Riyadh 11313, Saudi Arabia; shariqnajeeb@gmail.com; 3 School of Metallurgy and Materials, University of Birmingham, Birmingham B15 2TT, UK; drzohaibkhurshid@gmail.com; 4 Department of Biology, Faculty of Science, University of Isfahan, Isfahan 81746-73441, Iran; m.vaziri@sci.ui.ac.ir; 5 Department of Biomedical Engineering, College of Engineering, King Faisal University, Al-Hofuf 31982, Saudi Arabia; szohaib@kfu.edu.sa; 6 School of Dentistry, Riyadh College of Dentistry and Pharmacy, P.O. Box 84891, Riyadh 11313, Saudi Arabia; bilal.n.karshe@student.riyadh.edu.sa; 7 Department of Medical Engineering, University of Bradford, Bradford BD7 1DP, UK; F.Sefat1@Bradford.ac.uk or fsefat@kfu.edu.sa or fsefat@stevens.edu; 8 Department of Biomedical Engineering, King Faisal University, Al-Hofuf 31982, Saudi Arabia; 9 Department of Chemistry, Chemical Biology and Biomedical Engineering, Stevens Institute of Technology, Hoboken, NJ 07030, USA

**Keywords:** dentistry, dental materials, nanomaterials, nanotechnology, tissue engineering, regeneration

## Abstract

Electrospinning is a versatile technique that has gained popularity for various biomedical applications in recent years. Electrospinning is being used for fabricating nanofibers for various biomedical and dental applications such as tooth regeneration, wound healing and prevention of dental caries. Electrospun materials have the benefits of unique properties for instance, high surface area to volume ratio, enhanced cellular interactions, protein absorption to facilitate binding sites for cell receptors. Extensive research has been conducted to explore the potential of electrospun nanofibers for repair and regeneration of various dental and oral tissues including dental pulp, dentin, periodontal tissues, oral mucosa and skeletal tissues. However, there are a few limitations of electrospinning hindering the progress of these materials to practical or clinical applications. In terms of biomaterials aspects, the better understanding of controlled fabrication, properties and functioning of electrospun materials is required to overcome the limitations. More *in vivo* studies are definitely required to evaluate the biocompatibility of electrospun scaffolds. Furthermore, mechanical properties of such scaffolds should be enhanced so that they resist mechanical stresses during tissue regeneration applications. The objective of this article is to review the current progress of electrospun nanofibers for biomedical and dental applications. In addition, various aspects of electrospun materials in relation to potential dental applications have been discussed.

## 1. Introduction

Nanofibers remain an important division of biomaterials due to a wide range of biomedical applications [1]. The fabrication of nanofibers has attracted a lot of researchers due to unique properties required for biomedical applications for example availability of greater surface area for cellular interaction [2], protein absorption and binding sites to cell receptors [3]. Nanofibers can facilitate packing of maximum volume fraction by controlling fibers alignment and orientation hence improving the material strength [2]. The material properties such as surface morphology, porosity and geometry can be tailored or functionalized for certain applications, for example, bioactive agents for biomedical applications [4].

In order to fabricate fibers nanofibers, different techniques have been used for example, phase separation [5,6,7], nanofiber seeding [8] template synthesis [9,10], self-assembly [11,12] and electrospinning [13,14,15,16,17,18,19,20,21]. Amongst these techniques, electrospinning is a resourceful and cost effective technique that can be used to synthesize continuous nanofibers [1]. This technique can be used for soluble or fusible polymers alone or polymers can be modified with additives such as particles or enzymes to get the desired properties [20]. The resultant ultrafine fibers exhibit many interesting features, e.g., high surface area, tailorable porosity in the range of submicron to nanoscale and greater potential for surface functionalization [20,22,23,24]. In addition, electrospun fibers are considered an excellent candidate for a variety of biomedical purposes, e.g., wound dressings, drug delivery and tissue engineering scaffolds [21]. Electrospinning has been used for several biopolymers and blended biopolymers with synthetic polymers to obtain nanofibers [20]. Additionally, electrospinning can be used for fabricating polymer composite fibers by blending additives such as particles, antimicrobials or enzymes to get the desired properties [20].

Considering these benefits, electrospinning has gained a remarkable popularity for various disciplines hence, projecting a sharp rise in scientific publications in recent years (Table 1). There are relatively few electrospinning studies involving oral and dental sciences. The keywords “electrospinning” and “oral dental electrospinning” searched only 47 publications; 39 published in last five years (2011–2015) and only 8 published during 2005–2010. The aim of this article is to review the current progress of electrospun nanofibers for biomedical and dental applications. In addition, various aspects of electrospun materials in relation to potential dental applications have been discussed.

**Table 1:** Number of peer reviewed scientific papers published on “electrospinning” in recent years.

Years	Electrospinning		Oral/Dental Electrospinning
	Topic Search	Title Search	Topic Search
2005	296	114	1
2006	482	204	0
2007	623	259	0
2008	1047	373	1
2009	1183	442	2
2010	1431	507	4
2011	1845	579	3
2012	2102	627	12
2013	3377	639	8
2014	6117	793	10
2015	5233	600	6

Search was carried on using the keywords “electrospinning” and “oral dental electrospinning” in topic and title search options of ISI Web of Knowledge database for particular publication years.

## 2. Basic Principle and Technique

**Figure 1:**
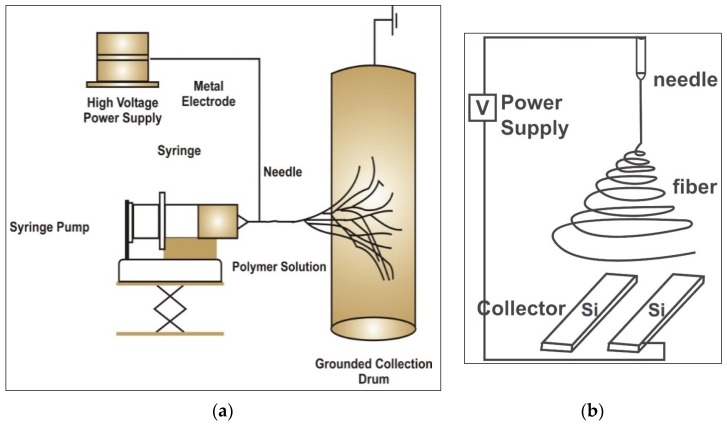
Schematic presentation of electrospinning process (**a**) a typical electrospinning equipment and its components (**b**) modifications of collector for aligning electrospun nanofibers.

The electrospinning technique involves the introduction of a strong potential difference between a polymer solution flowing through a capillary tip towords a metallic collector [25]. A typical electrospinning setup only requires a high voltage power supply, a syringe, a flat tip needle and a conducting collector (Figure 1a) [17]. However, the basic equipment can be modified for various applications such as using dual needle syringe (to make blended fibers), or rotating mandrel collectors (to make hollow tube like materials). Conventionally, electrospun materials have unwoven arrangement of nanofibers. Electrospinning with two strips of electrodes (Figure 1b) can be used to collect aligned fibers [26].

The potential voltage difference between the polymer solution and the collection plate, electrostatic forces overcome the solution surface tension to pull a jet of charged fluid that splits into nanofibers that fall towards the collection plate and solidify [27]. The polymer jet splits into multiple nanofibers that are deposited at the collector. The solvent evaporates as the jet is electrospun and leaving dry nanofibers on the collector [28].

## 3. Factors Affecting Electrospinning

Electrospinning is able to produce continuous nanofibers from a wide range of materials. However, there are many parameters (processing, physical, systemic and solution) which affect the fiber morphology and properties of electrospun fibers [29]. A list of key factors affecting electro-spun fibers is listed in Table 2 [25].

**Table 2:** List of variable parameter affecting the characteristics of electro-spun fibers.

Process Parameters	Systemic Parameters	Solution Parameters	Physical Parameters
Voltage	Polymer type	Viscosity	Humidity
Flow rate	Molecular weight	Concentration	Temperature
Collection plate	Polymer	Conductivity	Air velocity
Distance	Architecture	Dielectric constant	-
Angle	Solvent used	Surface tension	-
Motion	-	Charge of jet	-

### 3.1. Solution Related Parameters

The solution properties are important; it should have an optimal low surface tension and high enough charge density and viscosity so that collapse of the jet into droplets can be prevented before the solvent evaporates [30]. Polymer characteristics such as molecular weight, concentration, solution viscosity, surface tension and solution conductivity influence the nanofiber morphology and properties. Molecular weight represents the polymer chain length that in turn influences the entanglements; hence higher molecular weight results in viscous solutions compared to lower molecular weight. These entanglements prevent the jet from premature splitting during the process. Low viscous polymer solution jet breaks up into small droplets or creates beaded fibers [31]. Viscous solutions enhance chain entanglements and results in bead free uniform fibers. If, however, the viscosity is too high, it will be difficult to pump the solution through the capillary and the solution may dry up or drip at the tip [29].

Surface tension decreases the surface area of the solution and forces it to form a spherical droplet. In case of low concentration, high ratio of solvent molecules have greater tendency to assemble and form a spherical shape or bead formation [31]. In order to get bead free uniform fibers, low surface tension solvents should be used. In case of higher conductivity solutions (containing ions), the jet carries heavy amount of electrostatic charge. For example, adding a tiny fraction of salt or polyelectrolyte to electrospinning solution can increase the jet stretching and assists in forming smooth fibers in place of beaded fibers [32].

### 3.2. Polymer Concentration

The solution concentration below the threshold value will result in droplets formation instead of fibers. High solution concentrations result in viscous solutions and may lead to processing problems. For example, higher viscosity resists jet elongation and thinning and results in larger fiber diameter [33]. A previous study explored the relationship of polyethylene oxide (PEO) solutions viscosity and bead formation. Their results indicated that solution viscosity is linked to the bead size and density. Viscous solution resulted in less spherical and more spindle-like beads followed by nanofiber formation with occasional bead defects [31].

### 3.3. Processing Conditions

Processing conditions such as voltage, distance of collector, flow rate, needle guage and type of collector may affect the electrospinning process. High voltage induces required charges on the solution to cause the jet to emerge from the needle. Higher voltage accelerates more volume of electrospinning solution with relatively smaller Taylor cone [25]. Amount of solution available between the needle and electrospinning target is determined by the feed rate. The increase in voltage results in more stretching of solution and increased diameter due to the increase in feed rate. Increased feed rate may also cause fusing of fibers due to improper evaporation of solvent before the fiber is collected. The reduction in the distance causes shorter flight time for the jet. So the jet may not have enough time to solidify and result in fusing of fibers. Diameter of the orifice also has an effect. Smaller internal diameter reduces the clogging due to less exposure time of the jet to the environment. Reduction in needle internal diameter increases the surface tension of the solution corresponding to smaller droplet. This causes the acceleration of the jet to decrease. So jet gets more flight time before deposition and has more stretching and elongation; this results in smaller diameter fibers.

**Figure 2:**
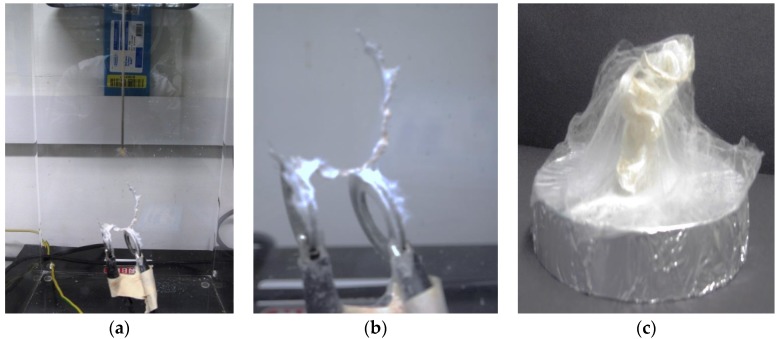
Fabrication of three dimensional (3D) electrospun scaffolds using regenerated natural *Bombyx mori* silk; (**a**) modification of electrospinning collector; (**b**) electrospun scaffold; (**c**) 3D natural silk electrospun using oval shape collector.

The above parameters are the major factors affecting the fiber morphology and web properties in electrospinning. Another factor is the design of the collector. Regular electrospinning yields randomly aligned nanofibers. Control on the geometry of deposition of fiber or getting other desired fiber patterns can be achieved with change in design of collectors. One of these is parallel bars with a gap between the two that leads to aligned nanofibers (Figure 2a). Li *et al.* [26] used this set-up for producing aligned fiber bundles. The fibers suspended across the nonmetallic part remain charged and align parallel due to repulsion between the electrospun and upcoming nanofibers [26,34].

### 3.4. Effect of Voltage

Increasing the applied voltage would discharge the polymer jet with stronger repulsion, causing it to undergo higher levels of drawing stress [33]. As a result there is a decrease in fiber diameter and therefore, the fiber diameter distribution would become increasingly higher, making the control of the process more difficult. An optimal voltage is required to initiate the polymer jet from the Taylor cone apex [35]. The applied voltage had a significant effect on droplet shape prior to jet formation. Higher voltage results in an increased flowrate of solution and faster electrospinning [25].

### 3.5. Volumetric Flow Rate

In order to stabilize the Taylor cone, the flow rate needs to be adjusted in a correct range. Vacuum usually form due to slow flow rate in the needle, causing the Taylor cone to disappear and temporarily stop the electrospinning process. Faster flow may buildup solution at the needle tip. As flow rate increases, the surface charge density decreases therefore the rate of charge withdrawal into the solution is dependent upon the residence time of ions in contact with the needle. The solution flow rate affects various features of nanofibers such as diameter, porosity, and geometry [25]. A constant and stable flow-rate is required to minimize the bead formation in electrospun materials [36]. Slow flow-rate reduced the diameter of electrospun nanofibers [37]. In addition, slow flow rate resulted in less number of beads and smaller diameter compared to faster flow rate [38].

### 3.6. Distance of Collector

It follows a negative power relationship as increasing the distance allows bending instabilities and whipping action to elongate and decreases the diameter of the polymer jet. There is a negative exponential relationship with surface charge density. Increasing gap distance drops the surface charge density. As the distance between the charged solution and collector increases, the magnitude of the electric field between the two decreases, forming fewer charged ions [39,40]. Another process parameter is the diameter of the needle tip as fiber diameter is reported to increase with a greater needle tip diameter [41,42]. In contrast, lack of correlation between needle diameter and resulting fiber diameter has been reported [43].

### 3.7. Effect of Conductivity

High conductivity enables polymer solutions to carry greater charge compared to low conductivity. Hence, high conductivity is yields greater tensile forces correspondingly to applied voltage and reduction in nanofiber diameter [44,45,46]. Fong *et al.* [31] examined the effect of sodium chloride to a polymer for electrospun nanofiber fabrication and reported a higher net charge density of the electrospinning jet. The increased charge density results in the formation of smooth and uniform nanofibers [31]. Zong and coworkers [45] explored the effects of adding salts to poly-DL-lactic acid (PDLLA) solutions and electrospun smooth, bead-free and fine diameter nanofibers. Alternatively, conductivity of polymer solution can be enhanced using surfactants [47,48]. Modifications using surfactants revealed similar results fabricating uniform and smaller diameter nanofibers [49,50].

### 3.8. Effects of Solvent

Solubility and boiling point of the solvent are important factors for choosing a solvent before electrospinning. Volatile solvents are ideal options due to rapid evaporation and dehydration of the nanofibers [51]. A very low boiling points favors rapid evaporation should be avoided to prevent the obstruction or occlusion of needle orifice prior to electrospinning. In contrast, high boiling points solvents may not dehydrate completely prior to hitting the target resulting in flat ribbon shape fibers instead of round fibers [50,52]. The volatility of the solvent may affect the microscopic features of electrospun fibers including porosity, shape and size hence, particular care must be taken during the evaluation and selection of electrospinning solvents [50].

## 4. Properties of Electrospun (ES) Materials

The electrospun materials may have unique chemical and physical properties distinguishing them from scaffold prepared using other fabrication techniques. Perhaps one of the most apparent advantages of electrospun scaffolds is ability to mimic extra-cellular matrix (ECM). It has been observed that cells seeded on highly porous electrospun meshes proliferate and differentiate at a higher rate when compared to less porous scaffolds [53]. Furthermore, *in vitro* studies conducted on nano-porous electrospun scaffolds strongly suggest that cells exhibit higher cellular adhesion with decreasing pore size and a higher pore density [54]. However, some studies suggest that fibers such as poly-L-lactic acid (PLLA), due to their hydrophobic nature, may have a detrimental effect on cellular adhesion. This can be overcome by spraying hydrophilic surfactants on such fibers [55]. Fiber orientation also plays a part in controlling the cellular growth. It has been seen that although osteoblast proliferation is somewhat comparable on aligned and random fibers, a higher calcium production has been detected when the cells are seeded on aligned fibers [56]. The generalized properties of electrospun tissue engineering scaffolds have been discussed here.

### 4.1. Physical Properties

Electrospun nanofibers can be fabricated in a range of diameters from micro to nanometers based on electrospinning process variables and modifications. The microscopic features of nanofiber are highly dependent on fiber morphology, diameter and surface area [57]. Nanofiber diameter is inversely proportional to surface area. The surface area facilitates cellular attachment and migration. Similarly, electrospun fiber reinforced epoxy composite materials showed improved toughness compared to unmodified resin composites [58]. Fibers also can attach to the surface in different orientation such as random, aligned or many specific shapes and patterns according to the underneath attaching surface. In tissue engineering, various electrospun nanofibers with various architectures and patterns employed for various tissues such as skin, bone and cornea [59,60]. Electrospun nanofibers may have infinite length and a random network of various lines corresponding to the longitudinal axes of fibers [61]. Pore size also plays a crucial role in cell attachment and cell infiltration in tissue engineering applications. Fiber diameter is an excellent indication of degradation in electrospun nanofibers [62].

### 4.2. Mechanical Properties

Mechanical properties of electrospun nanofibers are important as their applications in products should provide long life durability and structural integrity. Traditional testing methods can be applied for tensile testing of electrospun materials however, modelling and validating their behavior that requires the characterization of single a fiber is challenging. Mechanical properties of electrospun materials play a vital role and required to support cell growth and stability [63,64]. The poor mechanical properties and inability to manipulate certain mechanical properties for specific applications are real challenges for currently available electrospun materials. In order to improve the mechanical and handling properties of electrospun nanofibers, a number of techniques are employed. Cross-linking agents can be used to increase the tensile and flexural strengths of fibers [65]. Furthermore, scaffolds cross-linked with genepin display a better morphology after being immersed in water in addition to enhanced mechanical properties [66]. Conformational changes in polymers may be linked to mechanical properties. For example, β-sheet conformation in natural silk materials has better mechanical properties compared to α-helix conformation [67,68,69]. Further research is required to understand and improve the mechanical properties.

### 4.3. Biological Properties

Cell attachment to biomaterials especially electrospun nanofibers is crucial for tissue engineering applications. The major advantage of nanofibers is that they favor cell attachment because of higher surface area to absorb proteins and promoting binding sites [70]. Human embryonic stem cells showed promising results for cellular attachment while cultivated on polyurethane electrospun scaffolds [71]. Deshpande *et al.* [72] showed an excellent epithelial cell attachment to poly(lactic-*co*-glycolic acid) (PLGA) electrospun microfibers for cornea tissue engineering. Recently, a research group represented high cell attachment to Poly carpolactone (PCL)/Collagen electrospun nanofibers for skin tissue engineering [59]. Physical properties (pore size, volume) significantly affected the cell proliferation [73] and must be controlled during the assessment of biological properties. In addition to materials, cell electrospinning has been suggested as a valuable tool for functionalization of scaffolds for tissue engineering applications [74,75]. Electrospun fibers containing living cells for scaffold applications have been reported [75]. This approach can be used to fabricate a variety of biological (cellular) scaffolds using various cell lines and solvents. The final biological properties (such as cell count, type and medium) can be tailored depending on the type of target tissues and applications.

### 4.4. Chemical Properties

In the biological environment, degradable electrospun fibers are disintegrated chemically by enzymes such as lysozyme [76]. It is important to consider that there must not be any biocompatibility issues from broken down chemicals. In terms of tissue regeneration applications, the biodegradation rate should be controlled to match with the pace of tissue regeneration. The chemical properties of electrospun fibers is influenced by two main factors: hydrophilicity and chemical composition of the fibers. The electrospun fibers composed of copolymers have a reduced hydrophobicity and degradation compared to homopolymers [77]. Hence, altering the polymer chemistry may be an effective way to control the degradation rate of the fibers. As observed by You *et al.*, the crystallinity of polyglycolide, polylactide, and poly (lactide-*co*-glycolide) was decreased corresponding to *in vitro* degradation [78]. This accounts for the progress decrease in their mechanical properties. Indeed, the breakdown products of non-electrospun scaffolds such as poly-L-lactic acid (PLLA) and PLGA also account for the inflammatory response observed clinically [76]. However, there are insufficient randomized clinical trials to prove that similar reactions may be associated with electrospun scaffolds.

## 5. Electrospun Nanofibers for Dental Applications

**Figure 3:**
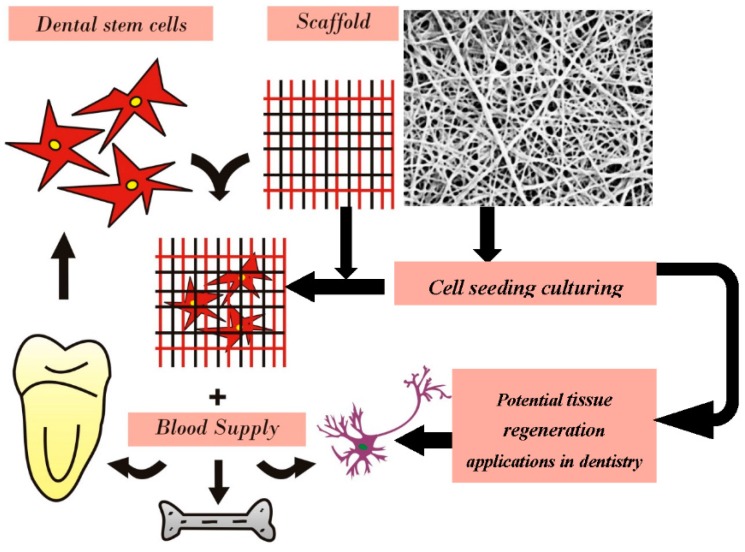
Schematic presentation of using electrospinning scaffolds for tissue engineering of various oral and dental tissues.

The major application of electrospun materials remain for tissue engineering and regeneration of oral and dental tissues. The electrospinning is an excellent technique for fabricating tissue engineering scaffolds [4,17,52,79]. A variety of materials including natural polymers (silk, collagen, chitosan), synthetic polymers (polyvinyl alcohol, polydioxanone) and nanocomposites (hydroxyapatite blends) have been electrospun for tissue engineering of oral and dental tissues (Table 3). In addition, these materials have been used for biomaterials applications such as modifications of implant surfaces, restorative nanocomposites and drug delivery. The typical approach for dental tissue regeneration using electrospun scaffolds is shown schematically in Figure 3. The progress and potentials of electrospun nanomaterials for dental applications has been discussed.

**Table 3:** Potential and progress of electrospun materials for dental applications.

Applications in Dentistry	Material(s) Electrospun	References
Tooth regeneration	Polyvinyl alcohol (PVA) Polydiaxonone (PDS)	[80,81,82]
Guided tissue regeneration	Collagen, Poly (lactide-*co*-glycolide) (PLGA) Poly-L-Lactic Acid (PLLA) Poly carpolactone (PCL) Polyethylene oxide (PEO), Silk	[83,84,85,86,87,88]
Caries prevention	Chitosan	[89]
Reinforcement of resin composites	Polyvinyl alcohol (PVA), Polyacrylnitrile, Polystyrene, Nylon	[90,91,92,93,94]
Implant modification	PLGA, Collagen	[95]
Cartilage regeneration	PCL Polyethylene oxide (PEO), Chitosan	[96,97,98,99,100]
Drug delivery	Poly(ethylene-*co*-vinylacetate), Poly(lactic acid) (PLLA), Poly (lactide-*co*-glycolide) (PLGA)	[77,101,102,103,104,105,106]
Wound and mucosal repair	Poly-L-(lactic acid), Poly (lactide-*co*-glycolide) (PLGA), chitin, chitosan, silk fibroin, collagen	[107,108,109,110,111]

### 5.1. Regeneration of Pulp Dentin Complex

Various pathological processes such as dental caries and trauma can result in the loss of dental tissues. Furthermore, various forms of pulp therapies are aimed at regenerating the roots of teeth (*apexogenesis*) that have undergone trauma. Although various medicaments such as calcium hydroxide, ferric sulphate and mineral trioxide aggregate are used as regenerative materials aimed at regenerating pulpal and radicular dentin, some cases still result in internal resorption of teeth [112,113]. In order to improve and speed up the results of pulp therapy, electrospun scaffolds have been studied that show the potential to induce odontoblast regeneration. Kim *et al.* produced electrospun scaffolds of polyvinyl alcohol and hydroxyapatite (HA) which could possess dentin regenerative properties [80]. Moreover, electrospun meshes of PCL have strongly shown potential for promoting odontogenic differentiation and growth as suggested by increased turnover of collagen I and other proteins when tested *in vitro* with human pulpal cells [81].

Endodontic therapy (root canal treatment) also requires delivery of drugs into the root canal and pulp chamber to eradicate the pathological microflora [114]. Bottino *et al.* produced electrospun scaffolds of polydiaxonone (PDS) in which antibiotics (metronidazole and ciprofloxacin) were incorporated in the solution. It was observed that these scaffolds were able to deliver the antibiotics more effectively and required a lower dose against pathogenic bacteria including *Porphyromonas gingivalis* and *Enterococcus faecalis* compared to drugs delivered via pastes [79]. Electrospinning has made it possible to produce bioactive 3D scaffolds made of PDS and halloysite aluminosilicate clay with the potential to regenerate pulp dentin complex by delivering agents such as antimicrobial drugs and growth factors [82]. Perhaps the biggest advantage of electrospinning is its ability to produce complex geometry of fibers to suit the specific application. The ultimate goal of regenerative endodontics is to regenerate the complex dentino-pulpal histology along with restoring the mechanical and physical attributes of the tooth. It is hoped that in the next decade electrospun fibers, along with injectable scaffolds and stem cells allowing optimum regeneration.

### 5.2. Guided tissue Regeneration for Periodontium

Untreated periodontal disease can lead to periodontal loss and eventual loss of teeth [115]. Regeneration of lost periodontal tissues had always been a challenge for clinicians. However, since advent of the guided tissue regeneration (GTR) membrane, it has been possible to regenerate lost bone by placing a barrier membrane between the gingival epithelium and the underlying periodontal bone [116]. Traditionally, non-resorbable materials such as expanded polytetrafluoriethylene (ePTFE) were used as GTR membranes but they had the disadvantage of requiring a secondary surgical procedure to remove the membrane which often carried a risk of infection and patient discomfort [76,116]. More recently synthetic and natural biodegradable materials such as collagen, Poly-L-Lactic Acid (PLLA) and PCL have been used as GTR membranes which degrade in the periodontal tissues thus avoiding the need of a second procedure to remove them [76,117,118,119]. Lately, electrospinning has been used to produce fibrous and porous electrospun biodegradable scaffolds as GTR membranes.

One of main advantages of electrospinning is its ability to produce fibers of different orientations and size for fibrous scaffolds for tissue regeneration [120,121]. Research indicates that these fibers are effective as tissue regenerative scaffolds because of their ability to mimic the fibrous extra-cellular matrix (ECM) of the human tissues such as bone and cartilage [122]. Indeed, it has been observed that a higher degree of fiber-orientation makes it possible to accelerate proliferation of fibroblasts. This has been attributed to an increased surface area and porosity of electrospun scaffolds [123]. Furthermore, changing the fiber orientation also makes it possible to “control” the direction of cellular proliferation as it has been that cells tend to proliferate in the direction of the fiber orientation [124,125].

The aforementioned properties of fibrous scaffolds can be taken advantage of if they are employed as periodontal GTR scaffolds. Many biodegradable materials have been electrospun and revealed the potential to function as GTR scaffolds [76,116]. Electrospun collagen nanofibers have the potential for GTR scaffolds applications [40]. Additionally, collagen fibers have the potential to allow differentiation of human bone marrow-derived mesenchymal stem cells (MSCs) [125]. However, to date, no studies have attempted to ascertain the mechanical properties of electrospun collagen fibers. Research has also been conducted to produce scaffolds composed of collagen blended with PCL, PEO, PLGA and PLLA [122,126]. One of the major disadvantages of collagen is that, due to its animal origins, there are ethical issues and concerns of cross-infection. Hence, the use of collagen scaffolds could be limited in quite a few demographics.

PCL is another material that has been electrospun to produce bone regenerative scaffolds [127]. It can be blended with collagen or other biodegradable polymers such as gelatin with enhanced tissue regenerative properties [128,129]. Moreover, biomimetic and osseoconductive materials such as nano-sized hydroxyapatite (nano-HA) crystals can be incorporated to PCL-PLA fibers to produce composite scaffolds [130]. Additionally, incorporation of nano-HA crystals not only increases the osteogenic potential of these scaffolds but it has also been suggested that these scaffolds have mechanical properties superior to those made of PCL alone [131]. Another exciting prospect of using electrospun scaffolds is their ability to function as carriers of growth factors and drugs such as bone morphogenic protein-2 (BMP2) and antibiotics which can enhance bone regeneration and prevent periodontal infections [83,84].

Although synthetic degradable polymers have been extensively studied to ascertain their periodontal regenerative properties, their major drawback is the production of acidic breakdown products resulting in inflammation at the site of implantation [76]. Hence, along with collagen, several natural polymers have been probed for GTR applications. Chitosan, a derivative of chitin which is a polymer present in the shells of crustaceans, can be electrospun to produce highly porous and fibrous scaffolds [85,86,87]. In order to improve the spinning ability and handling properties, chitosan was blended with PEO [132]. More recently, drug-incorporated and releasing chitosan-PEO fibrous scaffolds have been produced [29]. Natural silk is another example of degradable materials that has been electrospun for GTR and related applications [133,134]. Electrospun scaffolds of silk fibroin have shown promising results while human periodontal ligament (PDL) are seeded on their surface [15,17,18,135,136].

During the last few years, the idea of functionally graded membrane (FGM) has emerged [116,137]. This principle aims to produce a multilayered guided tissue regenerative membrane in which each layer has a specific function and physical properties, very much akin to the natural human tissues [138]. These layers can contain drugs and various growth factors which be released into the surrounding environment to enhance the regeneration of multiple tissues at the same time [139,140]. It has been speculated that electrospun fibers can form part of these FGMs [137]. Although electrospinning has added exciting new prospects to the field of guided tissue and bone regeneration, much more needs to be explored to validate the use of electrospun scaffolds in the clinical settings. For instance, more research is required to explore the mechanical properties of these scaffolds. More importantly, an adequate number of randomized clinical trials are required to prove their clinical efficacy.

### 5.3. Caries Prevention

Dental caries (tooth decay) not only lead to loss of tooth tissues but also have systemic ramifications. Conventionally, topical fluoride regimens in the form of mouthwashes, dentifrices [141,142,143,144] or fluoride-containing restorations [145,146] and oral hygiene measures have been used to prevent caries. More recently, electrospun mats having anti-caries properties have been studied. Non-toxic mucoadhesive chitosan fibers containing mangosteen extract have shown antibacterial activity against cariogenic pathogens including *Streptococcus mutans* and *Streptococcus sanguinis* [89]. These types of mats could be beneficial for individuals who are unable to self-administer oral hygiene protocols and can be used synergistically with existing methods.

### 5.4. Modification of Resin Composites

Like majority of polymeric materials, resin composites can be modified by addition of nano-sized fillers or nano-sized fibers [147,148,149]. Nanofibers produced by electrospinning have been incorporated to produce fiber-reinforced composites (FRCs). It has been observed that incorporation of nano-tube reinforced PVA fibers to resin composites can significantly improve the mechanical properties such as elastic modulus of resin composites [90]. However, dispersion of fibers leads to a decreased modulus due to weaker bonding between the fiber and resin phases. Electrospun polyacrylonitrile and polyamide containing nano-diamonds have shown to increase the mechanical properties of polymeric composites when fused with each other [91] that can be used as a means to reinforce dental composites. Similarly, incorporation of electrospun polystyrene fibers to epoxy has also been shown to improve the mechanical properties of the polymers [92]. Electrospun carbon nanotubes and nylon fibers have been successfully used to reinforce resin composites [93]. Production of self-healing nanofiber-reinforced resin composites holds an exciting prospect in increasing the marginal integrity and sealability of resin composites [94]. In addition to improving the mechanical and physical properties of resin composites and dental polymers, incorporation of electrospun fibers could also be used to produce bioactive composites and add anti-cariogenic properties to restorative materials. However, much needs to be learned about the bonding between the fibers and the composites as well as the biological and *in vitro* implications of these materials.

### 5.5. Implant Surface Modification

Dental implant is a surgical device that is in direct contact with the bone (*i.e.*, osseointegrated) and holds removable or fixed prosthodontic and orthodontic appliances [150,151]. Several materials (such as titanium and its alloys) have been used for dental implants. Recently, zirconia and reinforced polymers such as polyetheretherketone have also been used as dental implants [148,149,152,153,154,155,156]. To make the implant surface more bioactive and osseoconductive, several surface treatment methods have been employed [157,158]. However, many of these processes alter the mechanical or physical surface properties of the dental implant and ultimately leading to poor success rate [159,160]. To overcome these deleterious effects of surface modifications, several methods have been postulated but most of them are time consuming [158]. Electrospinning is an alternative attractive option that can be used to coat the implant surface using bioactive materials. In addition, electrospun nanofiber coatings have the benefits of greater surface area for the attachment of fibroblasts. Titanium dental implants coated by PLGA/collagen/nano-hydroxyapatite (n-HA) nanofibers significantly enhanced cellular proliferation on the surface and keeping water contact angles as low as 0° in addition to accelerated mineralization [95]. However, more studies are needed to investigate how well an electrospun coating adheres to a dental implant surface in comparison to conventional methods.

### 5.6. Cartilage Regeneration

Like elsewhere in the body, extensive trauma or pathologies in the head and neck region can result in the loss of not only the bone but also cartilage and ligament. Scaffolds hold potential to be a power adjunct tool to conventional surgical options. Electrospun PCL nanofibers have the potential to accelerate the proliferation of animal and human chondrocytes when tested *in vitro* [99,100]. PCL can be electrospun with fibrin to produce scaffolds of a combination of fibers having diameters in the range of both, nano- and micrometers to increase cellular infiltration [98]. Similarly, chitosan fibers have also shown potential to function as scaffolds for cartilage regeneration and they also possess mechanical properties superior to those of foams and hydrogels [97]. More recently a scaffold constructed of PCL, chitosan and PEO nanofibers has been used to successfully as a scaffold for chondrogenesis *in vitro* [96].

### 5.7. Drug Delivery

Like all other fields of surgery, dentistry requires preoperative and postoperative administration of drugs such as analgesics and antibiotics. As discussed above, electrospun scaffolds can be used as drug delivery devices to minimize the systemic dosage. Drugs such as antimicrobial agents, analgesics and anti-inflammatory regimens have been carried using electrospun scaffolds [77,105,106]. More recent applications of electrospun scaffolds include implantable drugs and growth factor-releasing scaffolds that help repair surgical sites by preventing infection and/or increase the rate of osseointegration [77,101,102,103,104,105,106]. PLLA fibers containing nanodiamonds loaded with growth factors not only possess better mechanical properties than unmodified fibers but can also carry and deliver growth factors and drugs to prevent infection, reduce inflammation and accelerate bone regeneration [161]. Furthermore, because the nanodiamonds can be made fluorescent, such scaffolds can be used to study and monitor guided tissue regeneration at a cellular level by using various imaging techniques [162,163].

### 5.8. Repair of Wounds and Oral Mucosa

Electrospun fibrous mats have been extensively researched as wound dressings capable of imparting anti-bacterial attributes to the wound as well as regenerative properties [107,108]. Such wound dressings can be used as media to deliver analgesics and antibiotics such topical anesthesia and which can decrease the amount of systemic administration of these drugs needed and hence help in decreasing their many unwanted adverse effects. Similarly, in dentistry, electrospun mats can used to deliver topical anesthesia and antibiotics to surgical or traumatic wounds [107]. In addition to wound repair, electrospun fibers could also be used as dressings for oral mucosal lesions such as ulcers or surgical wounds to relieve patients discomfort [109]. Polymers such as chitin and PLLA have been observed to function as effective scaffolds for proliferation and differentiations for human mucosal cells [109,110]. More recently, electrospun silk fibroin have also shown similar potential to human dermal matrices when tested against rat mucosal cells *in vitro* [111]. Although more studies are definitely required to ascertain the future of fibrous scaffolds and dressings for oral mucosal abrasions, these materials hold great promise in managing various mucosal ailments.

## 6. Limitations of Electrospinning

It is evident from the above discussion that electrospinning is a versatile technique that has fabricated unique materials for various biomedical and dental applications. However, there are a few limitations hindering the progress of its applications. Majority of biomedical and dental applications involve tissue engineering or regeneration hence, material’s ability to facilitate cell attachment and infiltration is very important. The randomly unwoven nature of electrospun mats and pore size does not provide ideal structure for cell infiltration [164,165]. Reducing the fiber dimeter increases the surface area however reducing the pore that may affect the cellular infiltration [57]. On the other hand, electrospinning is not a suitable technique for fabricating micron size or larger diameter fibers [1]. The fiber morphology can be altered however not very well controlled and is further complicated by the involvement of multiple electrospinning parameters. It has low fiber production efficiency [166].

Majority of tissue engineering applications required 3D scaffolds [164]. It is challenging to electrospun 3D scaffolds with precise dimensions and morphology [1]. Authors have attempted to electrospun 3D silk scaffolds successfully however, remain unable to control the dimensions and morphology precisely (Figure 2). In addition, there are a few technical challenges, for example, the rate of electrospinning. In order to electrospinning a couple of grams of polymer, it may take several hours. However, recent research suggested that incorporating salt solution (such as NaCl) results in thick meshes of 3D electrospun fibers [167]. Another way of using electrospun fibers and at the same time overcoming their mechanical shortcomings is making them components of multi-layered functionally graded membranes as proposed by Bottino *et al.* [103]. Additionally, incorporation of nanosized particles such as nanodiamonds has also been effective in increasing the mechanical properties of electrospun scaffolds [168].

Compared to cast membranes, electrospun materials are weaker mechanically. Detaching electrospun mats from the target substrate without damaging is a challenging task. Materials to be electrospun must be dissolved in a solvent of desired properties. The electrospinning solvents may alarm additional issues such as biocompatibility, pungent smell. Polymeric nature of materials restricts this technique for low concentration (~30% or below) solutions [169]. Another concern is the toxicity of the solvents and cross linking agents [170]. Although, relatively safer crosslinking agents such as genepin have been used recently, *in vivo* biocompatibility of these materials has yet to be evaluated extensively [171]. Hence, it is recommended that the electrospinning process should be carried in well-ventilated fume-cupboards with optimal conditions of humidity and temperature. Authors also recommend storing the polymer solutions in adequate conditions prior to electrospinning. It has been observed by the authors that chitosan-PEO solutions are very difficult to spin after being stored for 72 h. Therefore, the polymer solutions should be spun as soon as possible after being prepared Environmental conditions such as temperature and humidity also affect the electrospinning process [172].

Another important issue is the safety of the technical staff. Special precautions should be taken in order to avoid being electrocuted by the high voltage supply used for electrospinning. Therefore, insulating gloves and apparel in addition to protective masks should be worn. For tissue engineering and biological applications, the sterilization of materials is essentials. Special measures for sterilization must be considered to avoid any damages to the delicate nanofibers. These limitations are critical and may jeopardize the practical applications of electrospun nanomaterials. In order to translate electrospinning products from laboratory to clinical applications, further research is required to understand materials better and address these limitations.

## 7. Conclusions

There is no doubt that the electrospinning has gained popularity in recent years for bio-dental applications mainly for tissue engineering scaffolds. The progress of oral and dental tissue engineering is promising for the regeneration of oral tissues such as dentin, enamel, pulp, mucosa [173]. Various polymer and composite materials have been electrospun to fabricate scaffolds for tissue regeneration of dental tissues including dentin, periodontium, oral mucosa, bone and cartilage. The materials aspects of electrospun nanofibers such as fabrication, properties and functioning have already been explored in detail and suggested positive outcome for intended biomedical applications. The progress of electrospun materials for various oral applications is promising however there is lack of *in vivo* and clinical studies. There are a number of challenges (discussed in Section 6) that need to be resolved for further progress. Extensive research involving multiple disciplines (material scientists, chemists, engineers and health care professionals) is needed to translate the basic research to clinical trials and practical applications. It is very much expected that most of the limitations of electrospinning (Section 6) will be addressed in the near future and dragging electrospun materials for practical and clinical applications.
